# Women and Girls with Autism Spectrum Disorder

**DOI:** 10.1192/j.eurpsy.2022.207

**Published:** 2022-09-01

**Authors:** A. Potyrcha

**Affiliations:** Fondation Santé des Étudiants de France, Psychiatry, Paris, France

## Abstract

Autism spectrum disorder (ASD) is characterized by a triad of difficulties including altered communication, impairements in social internactions, as well as restricted interests and repetitive behaviours. It used to be known as a predominantly male disorder, but recent research has shown that the male/female ratio is trending downward and that the actual estimate is about 3:1. Until now, diagnostic criteria have been developed based on research conducted primarily in male populations. However, in recent years, female autism has attracted scientific interest, revealing some unique features in the presentation of this disorder in girls and women. This presentation will focus on gender differences and characteristics of ASD in females, including personal descriptions and experiences of women diagnosed with ASD.

**Disclosure:**

No significant relationships.

